# C1q-CD44 interactions regulate microglial phagocytosis, proliferation, and migration

**DOI:** 10.1186/s12974-026-03892-2

**Published:** 2026-06-12

**Authors:** Pooja S. Sakthivel, Alyssa J. Villegas, Anita Lakatos, Meghana Kaipa, Julian M. Lopez, Ashley Ling, Zeina H. Elrachid, Josh Karam, Aileen J. Anderson

**Affiliations:** 1 Sue and Bill Gross Stem Cell Research Center, University of California, Irvine, CA USA; 2 Department of Anatomy and Neurobiology, University of California, Irvine, CA USA; 3 Department of Physical Medicine and Rehabilitation, University of California, Irvine, CA USA; 4 Institute for Memory Impairments and Neurological Disorders, University of California, Irvine, CA USA

## Abstract

Microglia, the immune cells of the central nervous system (CNS), quickly respond to neurodegeneration by proliferating and migrating to areas of disease, phagocytosing debris, and releasing cytokines to initiate inflammation. Critically, the mechanisms underlying these microglial functions remain only partly understood. One molecular regulator of interest is complement protein C1q, the initiator molecule of the complement cascade that increases 300-fold in healthy aging and accumulates with neurodegeneration. We have previously reported that exogenous C1q treatment alters inflammatory gene expression and cell function in human induced pluripotent stem cell-derived microglia (iMG). Here, we test the hypothesis that C1q induced cell changes are modulated by novel C1q receptor, CD44. We first confirmed expression of five novel C1q receptors at the RNA and protein levels, and then validated C1q-receptor binding on the iMG cell surface using proximity ligation assay. Based on these results, we selected CD44 as an initial target and generated CD44 knockout iMG to test the role of CD44 in the iMG response to C1q. We demonstrate that C1q-CD44 interactions regulate changes in microglial phagocytosis, proliferation, and migration. These data suggest C1q interacts with CD44 to modulate microglial functions that are critical to health and disease, thus informing future directions to test whether these interactions are altered in neurodegenerative disease.

## Introduction

Aging and neurodegenerative disease trigger microglia, the resident immune cells of the central nervous system (CNS), to transition from a homeostatic state into an inflammatory state, which can have either beneficial or detrimental roles depending on the context. Critically, understanding the mechanisms regulating how microglia transition into an inflammatory state will inform therapeutic development for a wide range of neurodegenerative disorders.

One molecular regulator of interest is complement protein C1q, which is most traditionally known as the initiator molecule of the complement cascade. Upon recognition of an antibody or pathogen, C1q initiates the classical pathway through autocatalytic activation of C1 complex and consequential antibody-mediated killing of pathogens via a series of enzymatic reactions. However, in support of a novel role for C1q distinct from the cascade, local production of C1q is not always associated with terminal cascade proteins [17]. Microglia, for instance, locally produce early, but not late, components of the complement cascade in the CNS. Interestingly, C1q as a single ligand has been reported to polarize microglial state and drive secretion of inflammatory cytokines in primary rat microglia cultures, independent of the complement cascade [5]. We recently demonstrated that this phenomenon is conserved in human microglia, using recently developed protocols for robust and reproducible differentiation of human induced pluripotent stem cell (hiPSC)-derived microglia (iMG). In this paradigm, iMG display highly similar transcriptomic signatures to recently isolated primary human microglia, while also maintaining the heterogeneity in subpopulations characteristic of in vivo microglia [2, 12, 23], unlocking new ways to gain insights into human mechanisms of neuroinflammation. Using iMG, we have found that exogenous C1q treatment increases inflammatory gene expression and alters organelle and cell function [29].

In stark contrast to the traditional function of C1q in the complement cascade, these findings highlight the capacity of C1q to influence cellular behavior as a single ligand that is conserved across rodents and humans. Recent literature moreover supports a role for C1q as a single ligand in influencing neural stem cell fate and migration [3, 13], developmental synaptic plasticity [32], and astrocyte activation [20]. While increasing bodies of work suggest a distinct function of C1q in the CNS working as a single ligand, a mechanistic understanding of how C1q drives these complex changes in cellular function is currently lacking. In this regard, we have suggested that C1q can participate in receptor-mediated signaling and therefore conducted unbiased screening of plasma membrane proteins via C1q-pulldown and mass spectrometry in human neural stem cells [3]. This work identified five novel C1q receptors (ADCY5, BAI1, CD44, cMET, GPR62), and supporting the notion of C1q-receptor interactions, we have demonstrated that C1q binds to the transmembrane glycoprotein CD44 to regulate human neural stem cell migration [3].

Taken together, we hypothesized that C1q would similarly behave as a ligand with receptors on the microglial plasma membrane to regulate microglial inflammation. We utilized iMG to validate expression of all five candidate C1q receptors and their interactions with C1q on the microglial cell surface. After selecting CD44 as an initial target for investigation, we found that C1q-CD44 interactions modulate C1q-dependent changes in phagocytosis, proliferation, and migration. These data highlight a novel receptor-mediated mechanism underlying how C1q as a single molecule can influence microglial functions, suggesting that these interactions are relevant to inflammation and disease.

## Results

### Human microglia express candidate C1q receptors at the RNA and protein levels

**Fig. 1:**
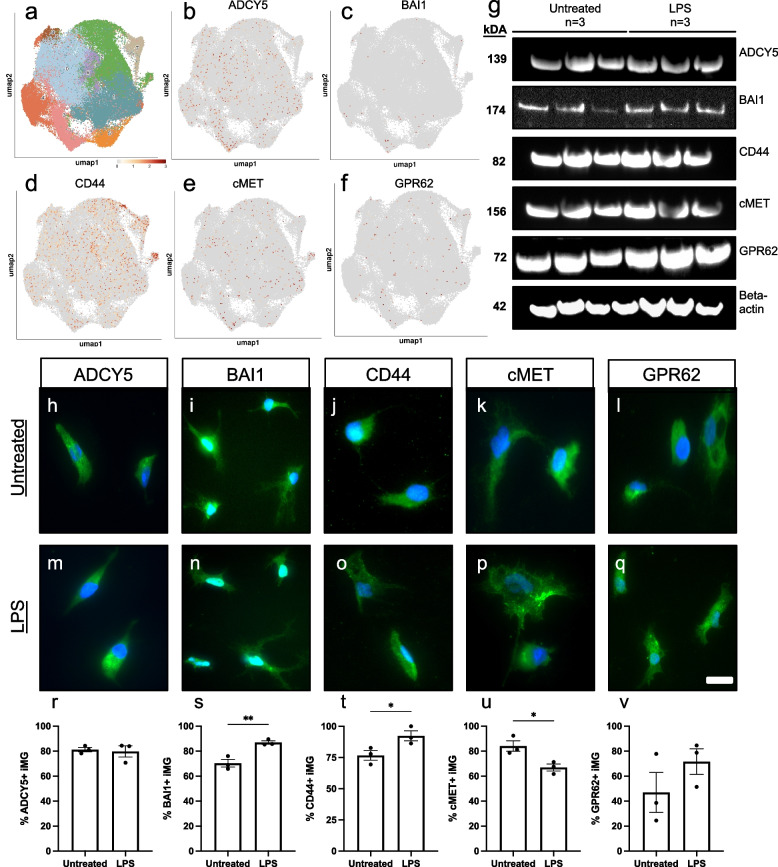
iMG express all five candidate C1q binding partners. Unbiased screening for C1q binding partners in human neural stem cells identified five novel signaling candidates in a previous study [3]. (**a**-**f**) Single cell RNA sequencing data of human microglia (**a**) confirms transcriptomic expression of ADCY5 (**b**), BAI1 (**c**), CD44 (**d**), cMET (**e**), and GPR62 (**f**). (**g**) Western blot validates expression of all five signaling candidates in untreated (*n*=3, left) and LPS-treated (*n*=3, right) iMG cell lysates. (**h**-**q**) Immunocytochemistry demonstrates positive expression of each target signaling candidate (green) and Hoechst (blue) in iMG. Untreated and LPS-treated iMG express ADCY5 (**h**,**m**), BAI1 (**i**,**n**), CD44 (**j**,**o**), cMET (**k**,**p**), and GPR62 (**i**,**q**). Scale bar = 10 μm. (**r**-**v**) Quantification of expression by immunocytochemistry in untreated and LPS-treated iMG for ADCY5 (**r**), BAI1 (**s**), CD44 (**t**), cMET (**u**), and GPR62 (**v**). Graphs represent quantification of the proportion of receptor+ cells in 6 random pictures/well. *n*= 3 biological replicates; mean ± SEM. Statistical analysis by Student’s T-test. *p≤0.05, **p≤0.01

We first tested whether the C1q candidate receptors are expressed in human microglia, in addition to neural stem cells, by evaluating a publicly-available integrated single-cell, human microglia RNA sequencing data set that combined 90,716 nuclei/cells across nineteen studies - need to add reference here - PMID: 39820004(Fig. 1A). These data validated transcriptomic expression of all five candidate receptors in human microglia (Fig. 1B-F). All candidate C1q receptors showed transcriptional expression, however, expression of CD44 was strikingly higher than other candidate receptors. Receptor expression patterns did not correlate with any of the nine subpopulations identified in this dataset, suggesting a pan-microglial role for C1q-receptor interactions. These data confirm that human microglia express all candidate receptors at the RNA level, and that CD44 expression is the highest.

Although ADCY5, BAI1, cMET and GPR62 showed lower transcriptomic expression patterns compared to CD44, we predicted that this could be due to stable protein expression on the cell surface, a particular feature for G protein-coupled receptors like BAI1 and GPR62. We therefore probed receptor expression at the protein level using western blot (Fig. 1G) and immunocytochemistry (Fig. 1H-Q). Cells remained untreated as a control or were treated with LPS, a commonly used stimulus that drives microglial inflammatory gene expression via activation of TLR4. We used western blot to confirm the presence of all candidate receptors at the protein level in untreated and LPS-treated microglial cell lysates collected after 24 h treatment (Fig. 1G). We additionally used immunocytochemistry to confirm expression of each candidate receptor at the single-cell level (Fig. 1H-Q). Quantification of the proportion of iMG expressing each receptor confirmed high expression of all five candidate receptors, and showed that LPS treatment increases BAI1 and CD44 expression, decreases cMET expression, and does not alter ADCY5 and GPR62 expression (Fig. 1R-V). Overall, these data validate expression of the candidate C1q receptors at the RNA and protein levels in microglia and could suggest that C1q acts as a ligand with these receptors, as it does in neural stem cells.

### C1q interacts with five novel C1q receptors on the microglial plasma membrane

**Fig. 2:**
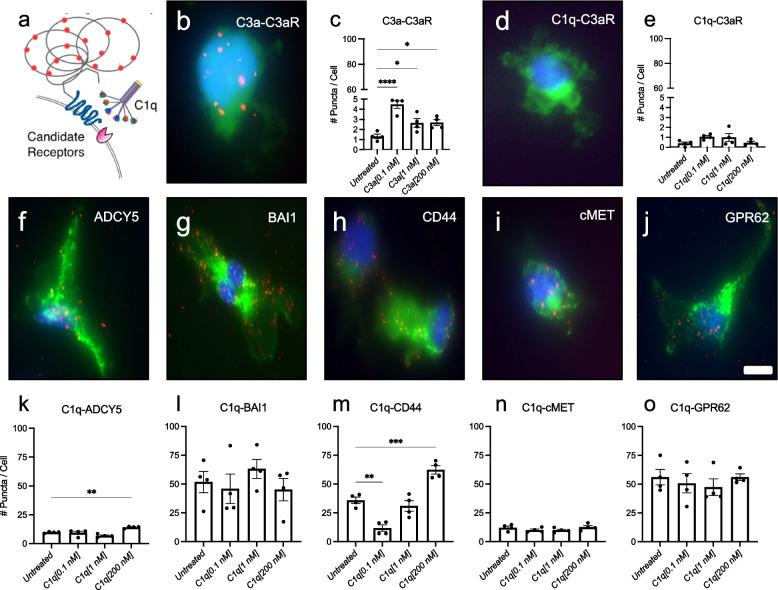
PLA verifies C1q-binding partner interactions at the microglial plasma membrane. **a** PLA shows quantifiable positive red fluorescent puncta when two target proteins are interacting within <30 nm. **b**-**e** PLA validation shows a representative image and quantification of a matched ligand-receptor positive control (C3a-C3aR) in (**b**-**c**) and a mismatched ligand-receptor negative control (C1q-C3aR) in (**d**-**e**). (**f**-**o**) PLA representative images and quantification of C1q with novel signaling candidates C1q-ADCY5 (**f**,**g**), C1q-BAI1 (**h**,**i**), C1q-CD44 (**j**,**k**), C1q-cMET (**l**,**m**), and C1q-GPR62 (**n**,**o**). Membrane staining (green) shown with Alexa 488 conjugated wheat germ agglutinin and nucleus shown with Hoechst (blue). Graphs represent quantification of the average number of fluorescent puncta/cell in 13 random pictures/well. *n*= 2 replicates (wells) were quantified across two independent experiments; mean ± SEM. Statistical analysis by one-way ANOVA. No significant difference in C1q-C3aR negative control, C1q-cMET, and C1q-GPR62 groups. All other groups showed significance for one-way ANOVA (*p*≤0.05) and analysis was followed by Dunnett’s multiple comparisons test as indicated. *p≤0.05, **p≤0.01, ***p≤0.001, ****p≤0.0001. Scale bar = 5 μm

Confirmation of receptor expression in microglia, however, does not mean that C1q plays a conserved role as a ligand for these receptors. Therefore, we next tested direct interactions between C1q and the candidate receptors on the microglial plasma membrane by employing proximity ligation assays (PLA), in which positive red fluorescent puncta indicate protein–protein interactions (Fig. 2A). To evaluate how C1q-candidate receptor interactions were influenced by exogenous C1q, we performed PLA following treatment with commercially available, purified C1q. To rule out the possibility that the obtained C1q could be aggregated or degraded, we performed SDS-PAGE and ponceau staining C1q to evaluate protein size. No differences in bands were identified between reducing and non-reducing conditions, demonstrating that protein aggregation was not as issue (Supp. Figure 1 A). These data confirm that commercially available C1q we obtained is not aggregated or degraded. Therefore, iMG were treated with purified C1q for 30 min at three physiologically relevant concentrations, as secreted by neutrophils (C1q[0.1 nM]), macrophages (C1q[1 nM]), or as present in the blood/serum (C1q[200 nM]) as previously described by our lab [13].

PLA validation was performed by imaging and quantification (the number of fluorescent puncta per cell) of a positive matched receptor ligand control (C3a-C3aR) (Fig. 2B, C) and a negative mismatched receptor ligand control (C1q-C3aR) (Fig. 2D-E). Figure 2F–J shows representative images of PLA detection of C1q-ADCY5, C1q-BAI1, C1q-CD44, C1q-cMET, and C1q-GPR62 interactions, respectively. Consistent with the fact that microglia are the largest producers of C1q in the CNS [7], we observed interactions between C1q and each candidate receptor in untreated (baseline) conditions in which no exogenous C1q was added. To quantify C1q secretion by iMG, we collected conditioned media from untreated and LPS-treated iMG and probed C1q concentration by ELISA. We found that iMG secrete C1q[0.25 nM] at baseline, and that this extracellular concentration is not altered by LPS at 24 hours post-stimulation (Supp. Figure 1B). These data confirm that iMG secrete C1q in vitro and that this endogenously produced autocrine C1q is available to interact with all five candidate receptors. Interestingly, all receptors showed different levels of baseline interactions with C1q in the untreated condition (Fig. 2 K-O). This data could be due to varying levels of each candidate receptor at the plasma membrane, or varying affinities between each receptor and C1q. Overall, we confirm that C1q interacts with these five candidate receptors on the microglial cell surface.

We next evaluated the dynamics of these interactions in response to C1q treatment, which we and others have shown induces microglial activation and polarization [5, 29]. We identified that the number of C1q-BAI1 (Fig. 2L), C1q-cMET (Fig. 2N) and C1q-GPR62 (Fig. 2O) interactions did not change in response to C1q treatment. These data suggest these receptors may have a low equilibrium dissociation constant and become saturated with autocrine C1q and/or C1q at low doses, or alternatively, that these interactions modulate a function that is not dependent on cell activation state. In contrast, C1q-ADCY5 (Fig. 2 K) and C1q-CD44 (Fig. 2 M) interactions showed an increase in puncta in response to treatment. These data suggest these receptors could be involved in a cellular function associated with the microglial state observed following C1q treatment. Validation of C1q interactions with these candidate receptors therefore reveals potential new mechanisms by which C1q may regulate microglia state and function. Critically, of the candidate receptors, CD44 displayed not only the highest level of transcriptomic expression (Fig. 1D), but also the most striking changes in number of interactions following C1q treatment (Fig. 2 M). We therefore selected CD44 for proof of concept analysis to investigate if the effect of C1q on microglia is dependent on a receptor-based mechanism.

### CD44 KO iMG display an altered transcriptomic profile, in comparison with WT iMG

With the goal to test how the response to C1q is altered without CD44, we generated wildtype (WT) and CD44 knockout (KO) hiPSC using CRISPR/Cas9 gene editing. Guide RNAs generated a 47 bp deletion in exon 2 of CD44 (Supp. Figure 2A-C) that led to a premature stop codon and successful generation of a knockout cell line, as validated by absence of CD44 expression in iMG via immunocytochemistry (Supp. Figure 2D-E) and western blot (Supp. Figure 2 F). Of note, CD44 is involved in numerous functions that are critical to microglia, such as neuroinflammation, migration, and cell–cell adhesion. CD44 also has multiple ligands produced by microglia themselves, including not only C1q, but also hyaluronic acid, osteopontin, and fibronectin. Thus, we anticipated that genetic disruption of CD44 would alter microglial transcriptional signature and function at baseline, even in the absence of stimulation with an exogenous ligand. However, we reasoned that these cells could still provide a tool to assess the response to C1q, so long as the capacity to respond to extracellular cues is intact. We thus first confirmed that generation of a CD44 KO did not exhibit a level of altered polarization and function that would prevent analysis of responses to C1q.

**Fig. 3:**
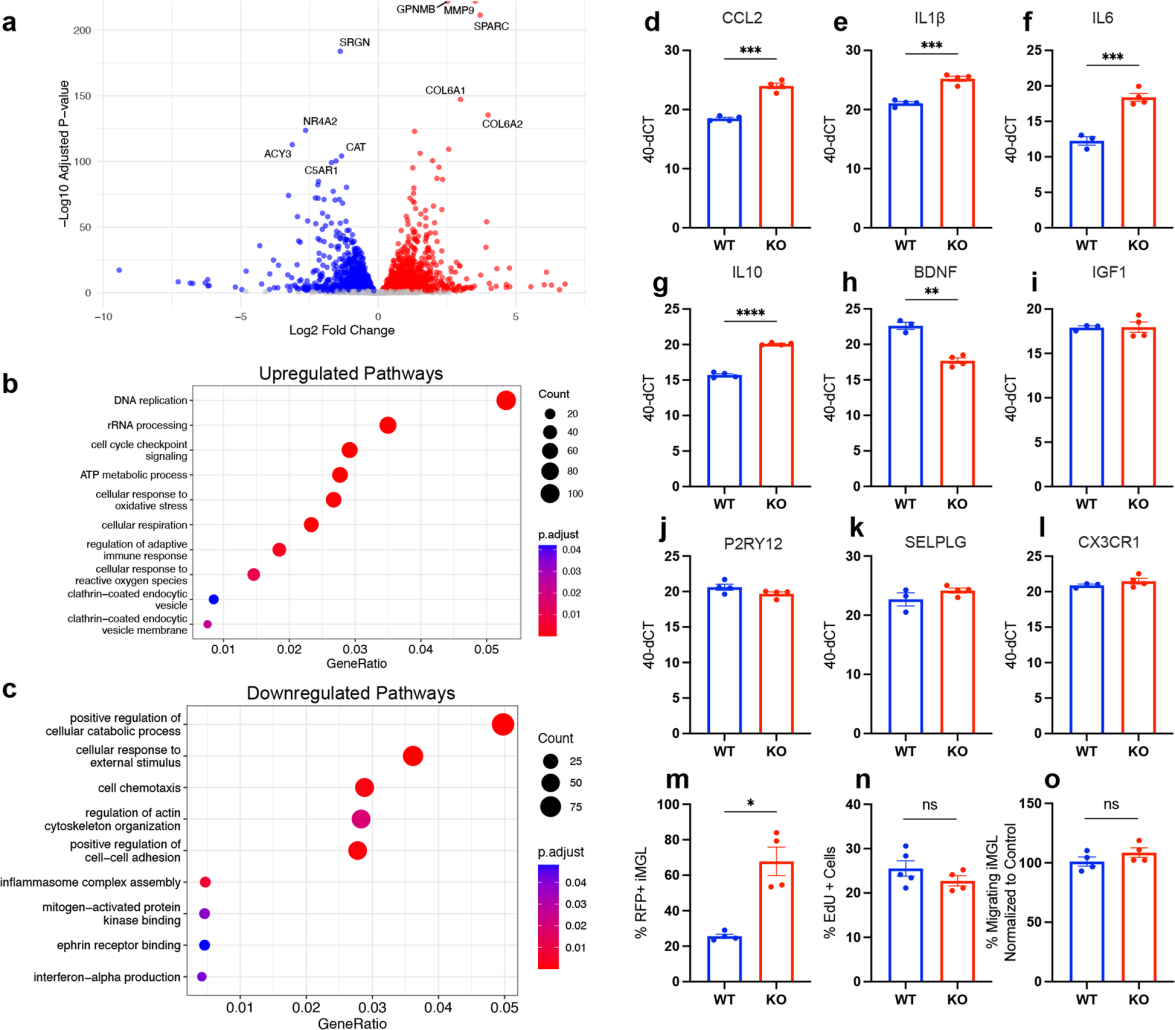
CD44 KO iMG display an altered transcriptomic signature and phagocytic baseline compared to WT iMG. **a** Bulk transcriptomics and differential gene expression analysis reveals many significantly upregulated (red) and downregulated (blue) genes when comparing WT and CD44 KO iMG. **b**-**c** Gene ontology by overrepresentation analysis reveals pathways that are significantly enriched (**b**) and repressed (**c**) in CD44 KO iMG, compared to WT. (**d**-**i**) qPCR analysis of WT vs KO cells shows changes in representative pro-inflammatory genes (**d**-**f**) and anti-inflammatory genes (**g**-**i**), but not change in homeostatic genes (**j**-**l**). (m) CD44 KO iMG display an increase in RFP, compared to WT iMG, following 1 h treatment with pHrodo particles, indicative of increased phagocytosis in KO cells. (**n**) WT and CD44 KO iMG both exhibit ~20–25% proliferation as quantified by EdU incorporation after 24 h and flow cytometry. (**o**) Transwell migration assay reveals that WT and CD44 KO iMG display similar migration profiles at baseline. **n**= 3–5 biological replicates; mean ± SEM. Statistical analysis by Student’s T-test. *p≤0.05, **p≤0.01, ***p≤0.001, ****p≤0.0001

As anticipated, bulk RNA sequencing revealed that CD44 KO iMG showed striking changes in RNA expression when compared to WT iMG, with 2,225 significantly upregulated genes and 2,110 significantly downregulated genes (Fig. 3A). Gene ontology by overrepresentation analysis revealed that CD44 KO iMG significantly upregulated genes related to DNA replication and cell cycle checkpoint signaling, consistent with the role of CD44 isoforms in numerous cancer types (Fig. 3B). CD44 KO iMG also exhibited upregulation of numerous pathways associated with pro-inflammatory or disease states, such as cellular response to oxidative stress, regulation of adaptive immune response, and cellular response to reactive oxygen species (Fig. 3B). We also observed an increase in clathrin-coated endocytic vesicle components (Fig. 3B), which are often altered in microglia in the context of neurodegeneration [33]. Increased expression of these pathways with no stimulus present implies that CD44 KO iMG are disrupted from homeostasis at baseline.

CD44 KO iMG, when compared to WT iMG, repressed cellular responses to external stimuli, as expected due to the genetic deletion of a receptor (Fig. 3C). CD44 KO iMG also decreased expression of cellular catabolic process, suggesting CD44 plays a role in autophagy, macromolecule turnover, or regulating energy production (Fig. 3C). We moreover observed that CD44 KO iMG downregulated cell chemotaxis, cytoskeleton organization and cell–cell adhesion pathways (Fig. 3C); again, this is consistent with the established role of CD44 in regulating migration and the extracellular matrix. Lastly, we found that CD44 KO iMG repressed inflammasome, MAPK, and interferon pathways (Fig. 3C). Decreased expression of these pathways again suggest that loss of CD44 expression alters homeostasis in microglia. We validated the bulk RNA sequencing findings by qPCR to demonstrate that CD44 KO iMG display disrupted homeostasis, as we quantified increased expression of CCL2, IL1β, IL6, and IL10, and downregulated expression of BDNF in CD44 KO iMG versus WT iMG (Fig. 3D-H). We also found that CD44 KO showed no change in expression of anti-inflammatory marker IGF1 (Fig. 3I), or homeostatic markers P2RY12, SELPLG, and CX3CR1 (Fig. 3 J-L). Overall, these data reveal that disruption of CD44 alters a number of inflammatory signaling pathways in iMG.

We considered the possibility that these transcriptomic changes are due to baseline disruption of CD44 interactions with autocrine C1q that is produced by microglia themselves. To test this hypothesis, WT iMG were treated with a C1q neutralizing antibody (NAb) for 24 h to block C1q function at baseline before bulk RNA sequencing. Our lab has extensively characterized this NAb in blocking C1q function, as previously published [13]. In stark contrast to the effect of CD44 KO, we found that treatment with C1q NAb in WT iMG for 24 h had a minimal influence on microglial transcriptional state, altering the expression of fewer than 100 genes (Supp. Figure 3 A). These data suggest that altered homeostasis due to CD44 KO is not dependent on secreted autocrine C1q in this paradigm.

We also evaluated whether the transcriptional changes identified in CD44 KO iMG were associated with baseline changes in function. WT and CD44 KO iMG phagocytosis was quantified by flow cytometry after a one hour incubation with pHrodo particles, which become fluorescent upon internalization into an endosome and indicate phagocytosis. CD44 KO iMG displayed increased phagocytosis rates when compared to WT iMG (Fig. 3 M), consistent with the interpretation that disruption of CD44 drives a pro-inflammatory microglial activation state. In contrast, CD44 KO iMG did not exhibit changes in proliferation via EdU incorporation and flow cytometry (Fig. 3N), or changes in migration via transwell migration assay when compared to WT iMG (Fig. 3O). These data show that, despite striking transcriptional changes and alterations in phagocytosis, CD44 KO iMG retain normal proliferation and migration profiles.

**Fig. 4:**
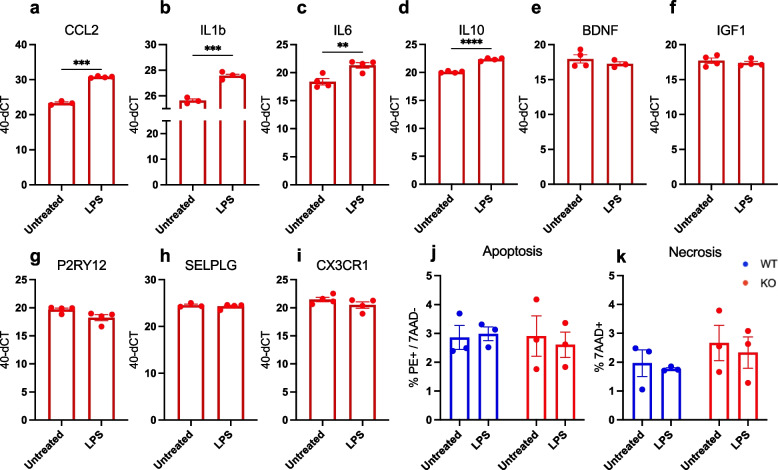
CD44 KO iMG retain the capacity to respond to LPS. (**a**-**i**) CD44 KO iMG remained untreated or were treated with 100 ng/mL LPS. Cells were lysed following a 24 hour treatment and RNA was collected to observe changes in representative inflammatory (**a**-**c**), anti-inflammatory (**d**-**f**), and homeostatic genes (**g**-**i**). LPS treatment drives an increase in CCL2, IL1β, IL6, and IL10 expression in CD44 KO iMG, suggesting there is no ceiling effect in inflammation in the KO. *n*= 3–4 biological replicates; mean ± SEM. Statistical analysis by Student’s T-test. **p≤0.01, ***p≤0.001, ****p≤0.0001. (**j**-**k**) Neither WT nor CD44 KO iMG show changes in apoptosis (**j**) or necrosis (**k**) at baseline or in response to LPS as shown by Annexin/7AAD expression quantified by flow cytometry. n=3 biological replicates; mean ± SEM. Statistical analysis using two-way ANOVA (*p*≤0.05), followed by Sidak’s multiple comparisons test as indicated. *p≤0.05, **p≤0.01, ***p≤0.001, ****p≤0.0001

Finally, we tested if CD44 KO microglia retained the capacity to respond to external stimuli, which is essential to enable study of C1q responses in these cells. Like WT iMG (Supp. Figure 4), CD44 KO iMG retained the capacity to respond to LPS, as quantified by increased CCL2, IL1β, IL6, and IL10 expression via qPCR (Fig. 4A-D). We observed no change in expression of representative anti-inflammatory (BDNF and IGF1) and homeostatic (P2RY12, SELPLG, CX3CR1) markers in response to LPS (Fig. 4E-I), suggesting the response to LPS is not fully intact. However, the robust increase in pro-inflammatory gene expression demonstrates that disruption of CD44 did not elicit a ceiling effect in activation state that would preclude the ability to detect further inflammatory modulation. Moreover, CD44 KO iMG do not show altered apoptosis or necrosis levels at baseline or in response to LPS (Fig. 4 J-K), highlighting that disruption of CD44 does not lead to premature cell death. These data demonstrate that, despite an altered baseline state, CD44 KO iMG can respond to extracellular cues and thus can be used to study the microglial response to C1q. We therefore next investigated how C1q influences microglial inflammation and whether this response is dependent on CD44.

### C1q modulation of the inflammatory transcriptional response is mediated in part through CD44

**Fig. 5:**
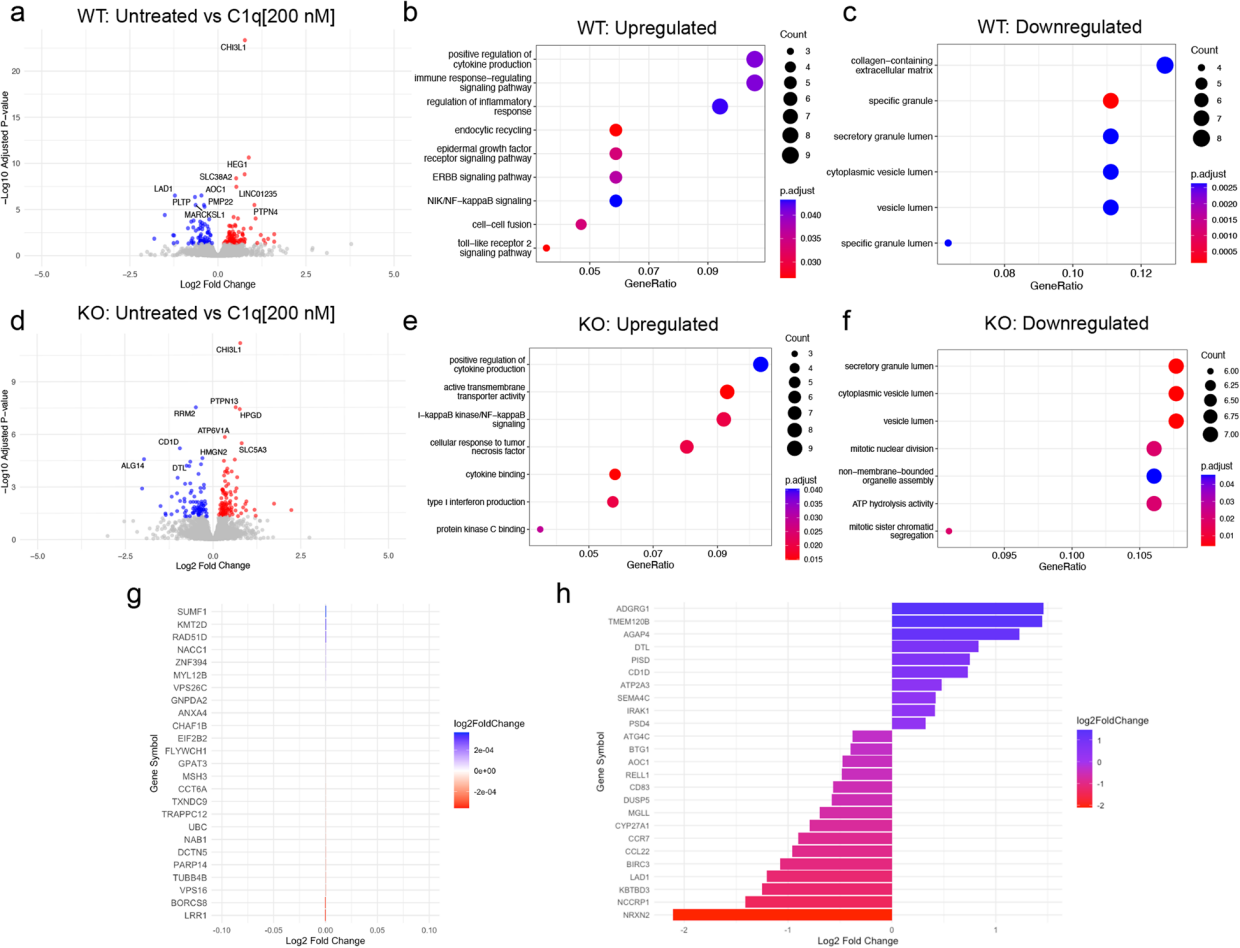
CD44 KO iMG do not transcriptionally respond to C1q as WT iMG do. (**a**-**c**) Differential gene expression of untreated WT iMG versus WT iMGL treated with C1q[200 nM] for 24 hours (**a**) and overrepresentation analysis highlighting enriched (**b**) and repressed (**c**) pathways in response to C1q treatment. (**d**-**f**) Differential gene expression of untreated CD44 KO iMG versus KO iMG treated with C1q[200 nM] for 24 hours (**d**) and overrepresentation analysis highlighting enriched (**e**) and repressed (**f**) pathways. (**g**-**h**) Barplots showed the least differentially expressed genes (**g**) and the most differentially expressed genes (**h**) when comparing the response to C1q in WT versus CD44 KO iMG

To test the hypothesis that CD44 is required for C1q-mediated changes in gene expression, we used bulk RNA sequencing to evaluate microglial transcriptional state in response to exogenous C1q treatment. WT iMG were treated with C1q[200 nM] for 24 hours, resulting in a significant upregulation of 89 genes and downregulation of 65 genes (Fig. 5A). Gene ontology analysis revealed that C1q enriched pathways were related to cytokine production and immune/inflammatory response in WT iMG. C1q also led to the upregulation of genes associated with EGF, ERBB, NF-κB, and TLR2 signaling pathways, all of which are associated with pro-inflammatory microglia (Fig. 5B). C1q moreover repressed pathways related to extracellular matrix and cellular lumen in WT iMG (Fig. 5C). These data together are consistent with the notion that C1q polarizes microglia to a pro-inflammatory state by driving cytokine expression and pro-inflammatory signaling pathways.

We next evaluated the effect of C1q treatment on microglial transcriptional state in CD44 KO iMG. CD44 KO iMG were treated with C1q[200 nM] for 24 hours, resulting in significant upregulation of 94 genes and downregulation of 72 genes (Fig. 5D). However, only 13/94 upregulated genes and 6/72 downregulated genes in the CD44 KO iMG response to C1q were conserved when compared to the WT iMG response to C1q, suggesting the transcriptional effect of C1q treatment is distinct between WT and CD44 KO iMG. While these data identify unique transcriptional programs between WT and KO iMG, the pro-inflammatory response to C1q remained largely intact at the pathway level. Indeed, C1q triggered a dramatic enrichment of positive regulation of cytokine production and NF-κB signaling in CD44 KO iMG, consistent with that observed in WT iMG (Fig. 5E). We validated this by qPCR, confirming that C1q treatment increased CCL2 and IL1β expression in CD44 KO iMG, as observed in WT iMG (Supp. Figure 5). C1q treatment also repressed pathways related to vesicle lumen, indicative of changes in organelle organization or secretory pathways, in both WT and CD44 KO iMG (Fig. 5F). These data highlight aspects of the C1q transcriptional response that are not dependent on CD44.

To probe what aspects of the C1q transcriptional response are dependent on CD44, we analyzed the interaction effect between genotype and treatment, extracting genes that exhibited the least versus most change between WT and CD44 KO iMG in response to C1q treatment as identified by log_2_ fold change. While C1q regulated many similar genes in both WT and CD44 KO (Fig. 5G), we identified 25 genes that exhibited a significant difference in expression when comparing the response of WT and CD44 KO iMG to C1q treatment (Fig. 5H). Indeed, these transcriptomic genes were accompanied by identified differences at the pathway level by gene ontology in WT versus KO iMG. In particular, CD44 KO iMG exhibited a unique upregulation of type I interferon production and protein kinase C binding in response to C1q, that was not observed in WT iMG (Fig. 5E). Because we observed both conserved (Fig. 5G) and unique (Fig. 5H) gene expression patterns between WT and CD44 KO, these data suggest that C1q induces a pro-inflammatory transcriptional program in microglia that is only partially modulated through CD44.

### CD44 regulates C1q-induced changes in phagocytosis, proliferation, and migration

Lastly, we tested the hypothesis that C1q-CD44 interactions modulate microglia at the functional level. We and others have previously shown that C1q treatment leads to increased microglial phagocytosis, decreased proliferation, and increased migration [29]. Consistently, the bulk RNA sequencing data generated in this study highlight differential expression of genes and pathways related to phagocytosis, proliferation and migration. One possibility is that C1q treatment alters the expression of CD44 ligands, indirectly regulating the functional cellular behavior. To test this hypothesis, we investigated the effect of C1q treatment on these ligands using the RNAseq dataset generated in this study. No changes in alternative CD44 ligand expression were identified (Supp. Figure 6A), thus excluding this possibility. We next sought to probe the functional consequences of direct C1q-CD44 interactions by testing the effect of C1q on microglial function in WT versus CD44 KO iMG.

**Fig. 6:**
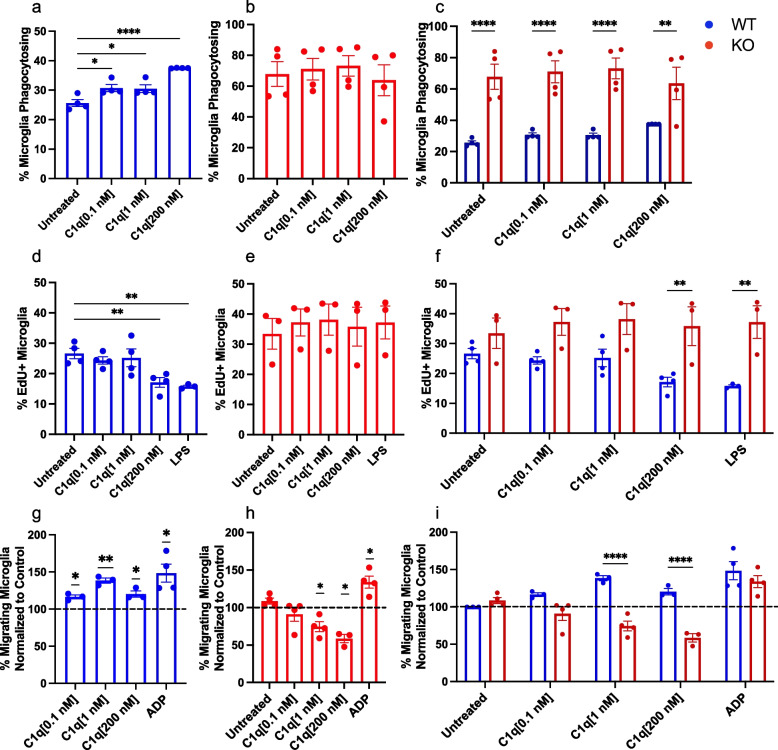
CD44 KO iMG do not functionally respond to C1q as WT iMG do. (**a**-**c**) C1q and pHrodo particle treatment, followed by quantification via flow cytometry, reveals that WT iMG increase phagocytosis in response to C1q treatment (**a**) but CD44 KO iMG do not (**b**). There is a significant difference in phagocytosis rates in WT vs KO iMG at baseline and across C1q treatments (c). (d-f) C1q and EdU treatment, followed by quantification via flow cytometry, reveals that WT iMG show a decrease in proliferation in response to C1q[200 nM] and positive control LPS (**d**). CD44 KO iMG, in contrast, do not decrease proliferation in response to C1q[200 nM] or LPS (**e**). There is a significant difference in proliferation rate between WT and KO iMG in response to C1q[200 nM] and LPS treatment (**f**).(**g**-**i**) Transwell migration assay following C1q or ADP treatment. All data was normalized to observed fluorescence in WT untreated condition. WT MG increase migration towards C1q and positive control, ADP (**g**). In contrast, CD44 KO iMG display decreased migration at C1q[1 nM] and C1q[200 nM], but retain increased migration towards ADP (**h**). WT vs KO iMG display significantly different migration effects in response to C1q[1 nM] and C1q[200 nM] (**i**). *n*=3–4 biological replicates; mean ± SEM. Statistical analysis by one-way ANOVA (*p*≤0.05), followed by Dunnett’s multiple comparisons test as indicated in a, b, d, e, g, h. Statistical analysis using two-way ANOVA (*p*≤0.05), followed by Sidak’s multiple comparisons test as indicated in c, f, and i. *p≤0.05, **p≤0.01, ***p≤0.001, ****p≤0.0001

It has been well-characterized that C1q can alter phagocytosis through both opsonization and other independent mechanisms [9]. Thus, we first assessed if C1q-mediated phagocytosis is dependent on CD44. We utilized conjugated pHrodo bioparticles that are non-fluorescent in neutral pH (i.e. cell media) but emit red fluorescent protein (RFP) once internalized into a phagosome that is pH ~4.5–5. This methodology allowed us to quantify RFP levels as an indirect readout of C1q-mediated phagocytosis. We tested the hypothesis that CD44 is essential for C1q-mediated phagocytosis by incubating WT and CD44 KO iMG with C1q[0–200 nM] and pHrodo particles for 1 hour, followed by quantification via flow cytometry. As expected, C1q triggered increased phagocytosis in WT iMG (Fig. 6A), but not in CD44 KO iMG (Fig. 6B). Notably, CD44 KO iMG display significantly higher phagocytosis rates at baseline, in comparison to WT iMG at baseline (Fig. 6C). To rule out the possibility that genetic disruption of CD44 elicited a ceiling effect in phagocytosis, WT and CD44 KO iMG were treated with LPS and pHrodo particles. We found that LPS triggeredincreased phagocytosis in both WT and KO iMG (Supp. Figure 7A-B), confirming CD44 KO iMG retain the capacity to increase phagocytosis in response to external stimuli. Therefore, significant differences observed between WT and KO iMG across all C1q doses suggest that CD44 is required for microglia to exhibit C1q-induced phagocytosis (Fig. 6C).

We next tested changes in microglial proliferation by EdU incorporation and flow cytometry after 24 hour treatment. C1q[200 nM] decreased proliferation in WT iMG (Fig. 6D), and this effect was dependent on CD44 expression, as it was lost in CD44 KO iMG (Fig. 6E). Because CD44 KO reversed the C1q-induced proliferation decline observed in WT iMG (Fig. 6F), we conclude that C1q-induced changes in proliferation require CD44.

We also tested the effect of LPS on microglial proliferation in WT and CD44 KO iMG. LPS has been noted to decrease proliferation in primary rat microglia [41], and we replicated this finding in our model. Interestingly, LPS binds to TLR4, which has been reported to generate a co-receptor complex with CD44 to modulate cell proliferation in non-immune cells [28]. These data suggest a potential role for CD44-TLR4 interactions in similarly mediating LPS-induced proliferation signals in microglia. In support of this notion, while LPS treatment decreased WT iMG proliferation, this effect was not observed in CD44 KO iMG (Fig. 6F). These data therefore suggest LPS-induced changes in proliferation are also dependent on CD44.

While one possibility is that LPS alters microglial proliferation via CD44-TLR acting in a co-receptor complex [37], another possibility is that LPS treatment alters autocrine C1q production by microglia and has downstream effects on cell proliferation via C1q-CD44 interactions. We thus quantified C1qa transcripts in response to LPS treatment by qPCR, which revealed that LPS triggers increased autocrine C1q production in iMG (Supp. Figure 6B). We next investigated the latter possibility by treating iMG with a C1q NAb ± LPS treatment and quantifying proliferation, testing whether LPS alters proliferation via autocrine production of microglial C1q. While C1q blockade via NAb decreased WT iMG proliferation rates at baseline, treatment with LPS in the presence NAb treatment did not reverse LPS-induced decreased proliferation (Supp. Figure 6 C). Critically, neither LPS nor NAb treatment altered apoptosis or necrosis rates in WT iMG (Supp. Figure 6D-E), identifying that these data reflect changes in proliferation and not cell viability. Together, these data suggest that LPS-induced proliferation changes are not dependent on autocrine C1q production by microglia.

We lastly probed a role for CD44 in modulating C1q-mediated chemotaxis. We and others have shown that microglia migrate towards C1q [18, 29]. Accordingly, we tested if this effect is dependent on CD44 using a transwell migration assay. While WT iMG migrate towards C1q at all tested doses (Fig. 6G), CD44 KO iMG exhibited decreased proliferation in response to C1q treatment (Fig. 6H). WT and KO iMG exhibited equivalent capacity to migrate towards ADP as a positive control, thus, decreased migration in response to C1q was not due to a migration deficit in CD44 KO iMG (Fig. 6I). These data demonstrate that C1q-CD44 interactions modulate microglial chemotaxis. In sum, our data demonstrate that C1q-CD44 interactions mediate microglial phagocytosis, proliferation, and migration, revealing a novel mechanism by which microglial function is regulated.

## Discussion

While CD44 has several ligands that have been well-studied (i.e. osteopontin, hyaluronic acid), here we investigate the relationship between CD44 and its most recently identified ligand, complement protein C1q [3]. In this study, we hypothesized that C1q signals through CD44 to modulate microglial function via receptor-mediated signaling. We first confirmed transcriptomic/proteomic expression of five recently identified C1q receptors in iMG, and then validated C1q-candidate receptor interactions using PLA. CD44 was identified as an initial target based on its high expression patterns and the striking increase in C1q-CD44 interactions following C1q treatment. Using CD44 KO iMG, we demonstrate that CD44 is required for microglia to exhibit C1q-induced changes in phagocytosis, proliferation, and migration. These data suggest that C1q-CD44 interactions modulate functions that are critical for microglia in both homeostasis and disease.

As microglia are constantly surveying their surroundings, the transition into disease-associated microglia (DAM) occurs quickly in response to local microenvironmental signals. DAM rapidly proliferate, migrate towards regions of disease, phagocytose debris and pathogens, and secrete inflammatory cytokines to facilitate neuroinflammation. Notably, one DAM marker that has been identified across numerous disease models is CD44. Microglial CD44 expression is elevated following stroke [30], ALS [30], glioma (., 2022), and Alzheimer’s disease (Rangaraju et al., 2018). CD44 + microglia/macrophages appear in the infarct region following ischemia and CD44 expression persists into the chronic phase post-stroke [30]. Both astrocytes and microglia display increased CD44 expression at disease onset that continuously increases in rodent models of ALS [22]. Microglia/macrophages within the glioma microenvironment also express high levels of CD44, and expression is correlated with tumor progression in humans [44]. Similarly, expression of CD44 is significantly elevated in post-mortem Alzheimer’s disease brain when compared to age-matched control tissue [25]. Interestingly, C1q expression dramatically increases 300-fold across healthy aging and further accumulates in neurotrauma and neurodegeneration [36]. This literature demonstrates the abundance of both C1q and CD44 in the disease microenvironment and highlights the potential for C1q-CD44 interactions to modulate inflammation via a conserved mechanism across neurodegenerative diseases and CNS cell types.

Indeed, numerous studies have found anti-CD44 antibody treatment inhibits inflammation in rodent models of arthritis (Zeidler et al., 1995), cutaneous inflammation (Camp et al., 1993), multiple sclerosis (Brocke et al., 1999), vascular leak syndrome (Rafi-Janajreh et al., 1999), and Parkinson’s disease (Wang et al., 2022). Moreover, blocking CD44 inhibits LPS-induced TLR4 expression and NF-κB p65 nuclear translocation in BV2 murine microglial cells (Wang et al., 2019). Conversely, CD44 stimulation via monoclonal antibodies or soluble hyaluronan triggers the release of inflammatory cytokines TNF-α and IL-1 in both human monocytes and murine macrophages (Webb et al., 1990; Noble et al., 1993). Therefore, CD44 has a very well-established role in regulating inflammation within the CNS.

Based on this data, we hypothesized that CD44 would similarly be required for C1q-induced neuroinflammation. In contrast, however, we found that CD44 KO iMG retain the ability to upregulate immune response genes following C1q treatment. Several possibilities may provide insight into this result. First, in contrast with our human microglia model, previous CD44 studies have primarily utilized rodent models or immortalized human cell lines. Indeed, human versus mouse microglia show varying expression of neurodegenerative ‘risk genes’, including complement proteins C1q and C3a, highlighting the importance of using human iMG to investigate cellular mechanisms as employed here [4, 10]. Second, previous studies have utilized antibodies or hyaluronan as stimuli, which may induce a distinct mechanistic response when compared to stimulation via C1q. In support of this notion, low versus high molecular weights of hyaluronan differentially interact with CD44 to drive changes in macrophage and endothelial cell function [16, 27]. In addition to these points, we note that disruption of CD44 by generation of KO iMG altered the baseline transcriptional program. It is likely that this is due to disruption of cell–cell and cell-extracellular matrix interactions, as CD44 is well known to act as an adhesion molecule [11]. Interestingly, while both WT iMG and CD44 KO iMG upregulate similar functional pathways in response to C1q, there is little overlap between the significant differentially expressed genes in WT versus KO iMG, suggesting that CD44 KO iMG exhibit conserved inflammatory responses but via distinct gene expression profiles.

While the transcriptional influence of C1q was not directly mediated by CD44, we did identify that C1q-CD44 interactions modulate functional changes that are critical to microglia, including phagocytosis, proliferation, and migration. While our data are consistent with the additional known roles for CD44, we identify for the first time a mechanistic understanding for how C1q modulates these functions in human microglia. CD44 is known to act as a phagocytic receptor in erythrocytes [40] and macrophages [39]. Consistent with the conventional role of C1q in opsonization for phagocytosis, our data suggest that C1q interacts with CD44 in microglia to facilitate debris and pathogen clearance. CD44 is also known to modulate proliferation of numerous cell types, including breast cancer cells [24], lung cancer cells [14], and epithelial cells [1]. We identify, for the first time, a function for CD44 in modulating proliferation in microglia in response to both C1q and LPS, and that this proliferation effect is not dependent on autocrine C1q production. Moreover, roles for both CD44 [6, 24, 43] and C1q [18, 29] in modulating migration have been established with limited mechanistic understanding. Here, we show for the first time that iMG migrate towards C1q in a CD44-dependent manner. These data together demonstrate C1q interacts with CD44 to modulate three critical microglial functions that are essential for health and disease: phagocytosis, proliferation, and migration.

While microglia are a subtype of macrophages and thus share many shared mechanisms of activation, the recent literature has drawn striking differences in microglia versus macrophage response to disease [26]. Interestingly, our data here identifying C1q as a regulator of microglial inflammation are in contrast with a set of literature investigating how C1q influences macrophage polarization. C1q has been shown to polarize both human monocytes and macrophages to an anti-inflammatory state via modulation of cytokine production [8, 34]. In contrast, we and others have demonstrated C1q polarizes microglia to a pro-inflammatory state [5, 29]. The doses of C1q employed in studies of both macrophages and microglia span the physiological range (C1q[0.1–300 nM]) and are overlapping, thus, a difference based on dose response is unlikely to underlie this observation. These data suggest that C1q may be one mechanism by which microglia and macrophages differentially modulate inflammation. However, the role of baseline activation state in these responses, particularly for *in vitro* models, must also be considered. Notably, C1q treatment in the presence of LPS suppresses LPS-induced inflammation in both microglia [5, 8] and macrophages [8]. These data suggest C1q may differentially modulate microglial state in the presence of other stimulation and highlight the interesting possibility that autocrine C1q, as produced by microglia themselves, may play a critical role in state regulation in the context of CNS pathogenesis.

It is also important to note that CD44 has numerous ligands, many of which are produced by microglia themselves and are potentially acting in an autocrine manner to self-regulate function. Interestingly, we found that transcriptomic expression of CD44 ligands was not altered following genetic disruption of CD44 at baseline. Similarly, neither WT nor CD44 KO iMG altered expression of alternative CD44 ligands in response to C1q treatment. While we found that autocrine C1q blockade with NAb did not alter iMG baseline transcriptional state or LPS-induced changes in proliferation, we did find that autocrine C1q is required for baseline iMG turnover, and that this function is CD44 independent. Future studies will investigate the four other C1q receptors whose expression profiles and interactions with C1q were validated in this manuscript.

Future work will also interrogate the downstream cellular pathways underlying CD44-dependent phenotypes. Indeed, CD44 has multiple known mechanisms of action, including regulating signaling cascades, cleavage of its extracellular and intracellular domains, and complexing with other cell surface receptors. Critically, the present study identifies a novel role for C1q-CD44 interactions in regulating microglial function. Interestingly, recent work suggests influences on microglial state and function by both genetics and sex [15, 19, 38, 42]. Expansion of this work to interrogate male and female patient lines, as well as the contributions of genetic factors in disease, will yield new mechanistic insights into how C1q-CD44 interactions modulate microglial function. In sum, we have identified a novel mechanism by which C1q directly modulates microglial behaviors through a recently identified receptor, CD44. These data provide the foundation to test if and how these mechanisms are altered in neurodegenerative disease, where both C1q and CD44 expression is elevated.

## Materials & methods

### hiPSC acquisition and maintenance

Human iPSC cell lines were generated by the University of California, Irvine Alzheimer’s Disease Research Center (UCI ADRC) Induced Pluripotent Stem Cell Core under approved Institutional Review Boards (IRB) and human Stem Cell Research Oversight (hSCRO) committee protocols. Non-integrating Sendai virus was used to perform reprogramming, thereby avoiding any integration-induced effects (Cytotune 2.0). iPSC were confirmed to be karyotype normal by G-banding, sterile, and pluripotent via trilineage in vitro differentiation. hiPSC were grown on 6-well plates that were pre-coated with Matrigel (1 mg/mL; Corning 356,231). Cells were maintained in a humidified incubator and received daily media changes with mTeSR Plus (Stem Cell Technologies 100–0276). During passaging, media was supplemented with 0.5 µM Thiazovivin (Stem Cell Technologies, 72,252). hiPSC were tested for mycoplasma every three months and confirmed to be negative.

### iMG generation

hiPSC were generated according to a previously published protocol [23]. Briefly, hematopoietic progenitors were made using STEMdiff Hematopoietic Kit (Stem Cell Technologies, 5310) and non-adherent CD43 + hematopoietic progenitors were collected at the end of the differentiation. Then, these cells were plated into iMG complete medium (DMEM/F12, 2X insulin-transferrin-selenite, 2X B27, 0.5X N2, 1X glutamax, 1X non-essential amino acids, 400 mM monothioglycerol, and 5 mg/mL human insulin freshly supplemented with 100 ng/mL IL-34, 50 ng/mL TGFb1, and 25 ng/mL M-CSF (Peprotech)) and fed every other day for 24 days. On day 25 and day 27, cells were fed iMG maturation medium (iMG complete medium, 100 ng/mL CD200 (Novoprotein), 100 ng/mL CX3CL1 (Peprotech),cells were ready for experimentation on day 28. Cells were incubated with purified C1q (MyBioSource, MBS147305) as indicated.

### Evaluation of public scRNA-seq data set

The publicly available scRNA-seq dataset from (Martins-Ferreira et al., 2025) used to visualize the expression of novel C1q receptors in human microglia.

### Western blot

Cell pellets were washed once with ice-cold PBS and lysed with RIPA buffer (Thermo, 89,900) containing protease (Roche, 5,892,970,001) and phosphatase inhibitors (Roche, 4,906,845,001) for 30 mn on ice with gentle agitation. Cell lysates were then centrifuged at full speed for 10 min at 4 °C, and supernatants containing isolated protein were collected into fresh tubes. Protein concentration was determined by BCA and equal amounts of total proteins were resolved in SDS-PAGE precast 4–12% gradient gels (Invitrogen, NP0321BOX) and transferred to PVDF membranes preactivated with methanol and equilibration buffer (GenScript, eBlot L1). PVDF membranes were reactivated with methanol and equilibration buffer following transfer, then blocked with BSA/TBST solution at 4 °C for 30 min. Receptors were probed by immunoblotting with primary antibody as indicated in Table i1 at 4 °C overnight orbital shaking. Following three TBST wash steps, bound antibodies were detected using species-specific HRP-conjugated donkey serum secondary antibody (Thermo, A16029). Secondary antibodies were incubated (1:5000) at room temperature for 1 h. Immunoblotting results were visualized using chemiluminescent ECL western blot substrate (Thermo, 34,075).

**Table 1:** Antibody applications and dilutions

Antibody	Species	Manufacturer,catalog #	Application
ADCY5	Rabbit	Abcam, ab66037	ICC: 1:100, PLA: 1:500, WB: 1:500
BAI-1	Rabbit	Abcam, ab135907	ICC: 1:1000, PLA: 1:500, WB: 1:1000
CD44	Rabbit	Abcam, ab51037	ICC: 1:1000, PLA: 1:500, WB: 1:1000
cMET	Rabbit	Abcam, ab5106	ICC: 1:500, PLA: 1:500, WB: 1:1000
GPR62	Rabbit	ThermoFisher, PA3048	ICC: 1:500, PLA: 1:500, WB: 1:1000
C1q	Mouse	Abcam, ab71940	PLA: 1:100
Beta actin	Rabbit	Abcam, ab8227	WB: 1:1000

### Immunocytochemistry

iMG were fixed in 4% paraformaldehyde for 15 min. Cells were blocked with PBS and 5% donkey serum for 10 min at room temperature. iMG were stained with primary antibodies as indicated in Table 1 and incubated for 2 h at room temperature. Cells were then washed with PBS 3 times for 5 min each. iMG were stained with secondary primary antibodies (Thermo Fisher, 711–606-152, 1:5000)and nuclear counterstain Hoescht (1:500) for 1 h at room temperature in the dark. iMG were then washed with PBS 3 times for 5 min each before cover slipping with fluoromount mounting solution.

### Proximity ligation assay

To validate cell surface C1q-receptor interactions, mature iMG were collected and plated on eight-well glass chamber slides in complete media (50,000 cells/well). Cells were incubated with purified C1q (MyBioSource, MBS147305) at concentrations of [0.1 nM], [1 nM], or [200 nM] for 30 min (full receptors) or 3 h (CD44 ICD) at 37 °C. Cells were then fixed with 4% PFA for 15 min at room temperature. For investigation of intracellular C1q-receptor interactions only, iMG were permeabilized using 3% Triton X-100 solution for 10 min at room temperature and followed by 3 PBS washes. Following fixation and/or permeabilization, cell cytoplasm was stained for 10 min at room temperature with wheat germ agglutinin (WGA, Alexa Conjugate 488, Invitrogen, W11261, 1:1000). PLA was then performed using the Duolink In Situ Red Starter Kit Mouse/Rabbit (Sigma-Aldrich, DUO92101) following the manufacturer protocol. This began by blocking the cells for 30 min at 37 °C using Duolink Blocking Solution. Primary antibodies indicated in Table 1 were incubated for 2 h at 37 °C and washed with Duolink in situ wash buffer A. Cells were then incubated with species-specific secondary antibody-complimentary PLA probes (1:5) for 1 h in humidified chambers at 37 °C. PLA probes were ligated for 30 min at 37 °C, then amplified for 100 min at 37 °C. Following amplification, the slides were washed with Duolink in situ wash buffer B and then cover-slipped using Duolink in situ DAPI mounting media (Sigma-Aldrich, DUO82040). For analysis, 12 random images per well were captured at 40 × using 0.275 µm intervals with a Zeiss microscope system. The number of red positive punctae was assessed manually using MBF Bioscience StereoInvestigator, providing an average fluorescent puncta count per cell. All experiments were performed in biological and technical duplicates.

#### ELISA

Conditioned media was collected from untreated and LPS-treated iMG after 24 h. C1q concentration was measured using an enzyme-linked immunosorbent assay kit for human C1q (Hycult, HK356). Samples were assessed in technical duplicate according to manufacturer’s instructions. Plate absorbance was recorded at 450 nm and samples were plotted against a standard curve to determine C1q concentrations.

### CD44 KO hiPSC generation

Ribonucleoprotein (RNP) delivery vectors were generated by first annealing Cas9 tracrRNA (IDTDNA, 1,072,533) and two customized gRNAs targeting exon 2 of CD44 at 95 °C for 10 min. Guide RNA 1 was 5' AATATAACCTGCCGCTTTGC 3' and guide RNA 2 was 5' TCGCTACAGCATCTCTCGGA 3’. The annealed gRNA was slowly cooled then complexed with HiFiCas9 enzyme (IDTDNA, 10,810,620) at a 1.2:1 molar ratio for 15 min at RT. hiPSC were dissociated by Accutase incubation for 1 min at 37 °C and resuspended in 65 µL nucleofection buffer (Lonza, VPH-5022) with Cas9 RNP and electroporation enhancer (STEMCELL Technologies) prior to nucleofection (Lonza, Amaxa Nucleofector program B-016). Transfected cells were plated in complete media containing mTeSR Plus (STEMCELL Technologies), 0.2 µm Thiazovivin (STEMCELL Technologies) and CloneR (STEMCELL Technologies) to recover for 24 h. Cells were then plated into as single cells for clonal selection by visual screening daily. DNA was isolated (Qiagen, 69,504) and the edit was validated by Sanger sequencing. Clones that passed screening were differentiated into microglia and knockout was again validated by immunocytochemistry and western blot.

### RNA Isolation and RT-qPCR

RNA isolation and RT-qPCR were conducted as described in [29]. Cells were harvested and lysed 24 h post treatment utilizing the Qiagen RNeasy micro extraction kit (Qiagen, 74,004). Initial lysis and protein denaturation were conducted using the RLT master mix with 1% β-mercaptoethanol followed by DNAse I treatment to degrade residual genomic DNA. Purified RNA were then reverse-transcribed into complementary DNA (cDNA) using commercially available high-Capacity RNA-to-cDNA kit (Applied Biosystems, 4,387,400). Integrity and purity of RNA samples were verified using a nanodrop spectrophotometer. RT-qPCR reactions were performed in 384 well qPCR plates at 10µL volumes using curated panel of primers listed in Table 2. Reaction master mix consisted of 2X TaqMan Gene Expression Fast Master Mix (Applied Biosystems, 4,444,557), 20X TaqMan Gene Expression primers, template cDNA, and molecular grade RNase free water. PCR plate was sealed and centrifuged prior to loading into Quantstudio7 Flex. Relative gene expression was determined utilizing the comparative ∆∆Ct method [31]. All experiments were performed with 3–4 biological replicates and 3 technical replicates.

**Table 2:** Primer assay IDs used for qPCR

Gene	Assay ID
18S	Hs03003631_g1
CCL2	Hs00234140_m1
IL1β	Hs01555410_m1
IL6	Hs00174131_m1
IL-10	Hs00961622_m1
BDNF	Hs02718934_s1
IGF1	Hs01547656_m1
P2RY12	Hs01881698_s1
SELPLG	Hs05033974_s1
CX3CR1	Hs01922583_s1

### Bulk RNA sequencing

RNA integrity (RIN) values and concentrations were determined using the Agilent Bioanalyzer 2100 series and Qubit respectively. KAPA HyperPlus Kit (Roche) was used for library construction via poly-A enrichment to enrich selection of mRNAs. Quality of DNA libraries and concentrations were determined using high sensitivity DNA assay on the aforementioned Bioanalyzer and Qubit. DNA libraries were quantified using Kapa PCR, normalized to 2 mM and then multiplexed for sequencing. Illumina HiSeq 4000 platform was used for sequencing with single read 100 base chemistry. Quality control was performed using FASTQC (Andrews, 2014) to validate the quality of sequencing files prior to alignment. Reads were then pseudoaligned to the human genome using Salmon, and gene level summary of transcripts were obtained via tximport [35]. Genes with at least 10 read counts in ≥ 3 samples were retained for differential expression analysis using DESeq2 [21] and an adjusted *p*-value (Benjamini–Hochberg) cutoff of 0.05. Volcano plots were generated using the “ggplot” function. Gene Ontology (GO) enrichment analysis was conducted using clusterProfiler [45]. All bulk RNA-seq analysis was performed in R programming language 4.2.1.

### Flow cytometry

Phagocytosis: Microglial phagocytic activity was measured through pHrodo ingestion, which exhibits a red fluorescence in response to acidity. iMG were simultaneously treated with C1q and 1:1000 pHrodo (Thermo Fisher, A10010) and incubated for 1 h at 37 °C. Cells were collected and washed twice with PBS/1% FBS before resuspension in 200 μL PBS/1% FBS prior to acquisition. Proliferation: Cell proliferation was assessed using flow cytometric detection of EdU incorporation. iMG were incubated with EdU (Cayman, 20,518) 1:1000 for 24 h in combination with treatment in the incubator. iMG were collected and fixed with 4% PFA for 15 min prior to detection with the Click- iT™ Plus Alexa Fluor™ 488 Picolyl Azide Toolkit (Invitrogen, C10641) according to the manufacturer's instructions. Apoptosis/Necrosis: Apoptosis and necrosis were assessed using PE Annexin V Apoptosis Detection Kit with 7- AAD (Biolegend, 640,934) according to the manufacturer's instructions. All samples were acquired on the BD Fortessa and data were analyzed using FloJo. All experiments were conducted in biological triplicate and technical triplicate.

### Migration assay

Migration assay was performed using migration assay kit (Sigma, ECM512) as previously described [29]. Briefly, cells were plated as 4 independent treatment replicates in a 96 well plate with transwells containing 0.5 µm pores. Cells were lysed after a 4 h incubation at 37˚C, labeled with fluorophore, and transferred to a black clear bottom 96 well plate for subsequent plate readout. All experiments were performed in biological and technical replicates.

### Statistical analysis

All experiments were performed with 2–4 biological replicates and 3–4 technical replicates depending on the assay. Gene expression analysis via qPCR was tested for statistical significance with either a one-way ANOVA with a post-hoc Dunnett’s multiple comparisons test or a Student’s t-test. Migration, proliferation, and phagocytosis was tested for statistical significance with either a two-way ANOVA with a post-hoc Sidak’s multiple comparisons test or a Student’s t-test. Proximity ligation assay quantification was tested for statistical significance using a one-way ANOVA with a post-hoc Tukey’s multiple comparisons test.

## Data Availability

Data is provided within the manuscript or supplementary information files.

## Abbreviations

CNS: Central nervous system
hiPSC: Human induced pluripotent stem cell
iMG: Induced pluripotent stem cell-derived microglia
LPS: Lipopolysaccharide
PLA: Proximity ligation assay
WT: Wildtype
KO: Knockout
NAb: Neutralizing antibody
DAM: Disease Associated Microglia

## References

[1] Abbasi AM, Chester KA, Talbot IC, Macpherson AS, Boxer G, Forbes A, et al. CD44 is associated with proliferation in normal and neoplastic human colorectal epithelial cells. Eur J Cancer. 1993;29(14):1995–2002. 10.1016/0959-8049(93)90461-N. 10.1016/0959-8049(93)90461-n 8280495

[2] Abud EM, Ramirez RN, Martinez ES, Healy LM, Nguyen CHH, Newman SA, et al. iPSC-derived human microglia-like cells to study neurological diseases. Neuron. 2017;94(2):278-293.e9. 10.1016/j.neuron.2017.03.042. 28426964 10.1016/j.neuron.2017.03.042 PMC5482419

[3] Benavente F, Piltti KM, Hooshmand MJ, Nava AA, Lakatos A, Feld BG, et al. Novel C1q receptor-mediated signaling controls neural stem cell behavior and neurorepair. Elife. 2020;9:e55732. 10.7554/eLife.55732. 32894219 10.7554/eLife.55732 PMC7476762

[4] Burns TC, Li MD, Mehta S, Awad AJ, Morgan AA. Mouse models rarely mimic the transcriptome of human neurodegenerative diseases: a systematic bioinformatics-based critique of preclinical models. Eur J Pharmacol. 2015;759:101–17. 10.1016/j.ejphar.2015.03.021. 25814260 10.1016/j.ejphar.2015.03.021

[5] Färber K, Cheung G, Mitchell D, Wallis R, Weihe E, Schwaeble W, et al. C1q, the recognition subcomponent of the classical pathway of complement, drives microglial activation. J Neurosci Res. 2009;87(3):644–52. 10.1002/jnr.21875. 18831010 10.1002/jnr.21875 PMC2635544

[6] Fernández-Tabanera E, García-García L, Rodríguez-Martín C, Cervera ST, González-González L, Robledo C, et al. CD44 modulates cell migration and invasion in Ewing Sarcoma cells. Int J Mol Sci. 2023;24(14):11774. 10.3390/ijms241411774. 37511533 10.3390/ijms241411774 PMC10381016

[7] Fonseca MI, Chu S-H, Hernandez MX, Fang MJ, Modarresi L, Selvan P, et al. Cell-specific deletion of C1qa identifies microglia as the dominant source of C1q in mouse brain. J Neuroinflammation. 2017;14(1):48. 10.1186/s12974-017-0814-9. 28264694 10.1186/s12974-017-0814-9 PMC5340039

[8] Fraser DA, Bohlson SS, Jasinskiene N, Rawal N, Palmarini G, Ruiz S, et al. C1q and MBL, components of the innate immune system, influence monocyte cytokine expression. J Leukoc Biol. 2006;80(1):107–16. 10.1189/jlb.1105683. 16617157 10.1189/jlb.1105683

[9] Galvan MD, Greenlee-Wacker MC, Bohlson SS. C1q and phagocytosis: the perfect complement to a good meal. J Leukoc Biol. 2012;92(3):489–97. 10.1189/jlb.0212099. 22715140 10.1189/jlb.0212099

[10] Geirsdottir L, David E, Keren-Shaul H, Weiner A, Bohlen SC, Neuber J, et al. Cross-species single-cell analysis reveals divergence of the Primate microglia program. Cell. 2019;179(7):1609-1622.e16. 10.1016/j.cell.2019.11.010. 31835035 10.1016/j.cell.2019.11.010

[11] Goodison S, Urquidi V, Tarin D. CD44 cell adhesion molecules. Mol Pathol MP. 1999;52(4):189–96. 10.1136/mp.52.4.189. 10694938 10.1136/mp.52.4.189 PMC395698

[12] Hasselmann J, Coburn MA, England W, Figueroa Velez DX, Kiani Shabestari S, Tu CH, et al. Development of a chimeric model to study and manipulate Human Microglia in vivo. Neuron. 2019;103(6):1016-1033.e10. 10.1016/j.neuron.2019.07.002. 31375314 10.1016/j.neuron.2019.07.002 PMC7138101

[13] Hooshmand MJ, Nguyen HX, Piltti KM, Benavente F, Hong S, Flanagan L, et al. Neutrophils induce astroglial differentiation and migration of Human Neural Stem Cells via C1q and C3a synthesis. J Immunol. 2017;199(3):1069–85. 10.4049/jimmunol.1600064. 28687659 10.4049/jimmunol.1600064 PMC5523578

[14] Hu B, Ma Y, Yang Y, Zhang L, Han H, Chen J. CD44 promotes cell proliferation in non-small cell lung cancer. Oncol Lett. 2018;15(4):5627–33. 10.3892/ol.2018.8051. 29552200 10.3892/ol.2018.8051 PMC5840516

[15] Kang S, Ko EY, Andrews AE, Shin JE, Nance KJ, Barman PK, et al. Microglia undergo sex-dimorphic transcriptional and metabolic rewiring during aging. J Neuroinflammation. 2024;21(1):150. 10.1186/s12974-024-03130-7. 38840206 10.1186/s12974-024-03130-7 PMC11155174

[16] Karam J, Singer BJ, Miwa H, Chen LH, Maran K, Hasani M, et al. Molecular weight of hyaluronic acid crosslinked into biomaterial scaffolds affects angiogenic potential. Acta Biomater. 2023;169:228–42. 10.1016/j.actbio.2023.08.001. 37572983 10.1016/j.actbio.2023.08.001 PMC11729822

[17] Kouser L, Madhukaran SP, Shastri A, Saraon A, Ferluga J, Al-Mozaini M & Kishore U. Emerging and Novel Functions of Complement Protein C1q. Front Immunol. 2015; 6. 10.3389/fimmu.2015.00317. 10.3389/fimmu.2015.00317 PMC4484229 26175731

[18] Lefebvre C. Calreticulin contributes to C1q-dependent recruitment of microglia in the leech *Hirudo medicinalis* following a CNS injury. Med Sci Monit. 2014;20:644–53. 10.12659/MSM.890091. 24747831 10.12659/MSM.890091 PMC3999160

[19] Li X, Li Y, Jin Y, Zhang Y, Wu J, Xu Z, et al. Transcriptional and epigenetic decoding of the microglial aging process. Nat Aging. 2023;3(10):1288–311. 10.1038/s43587-023-00479-x. 37697166 10.1038/s43587-023-00479-x PMC10570141

[20] Liddelow S, Guttenplan K, Clarke L, et al. Neurotoxic reactive astrocytes are induced by activated microglia. Nature. 2017;541:481–7. 10.1038/nature21029. 10.1038/nature21029 PMC5404890 28099414

[21] Love MI, Huber W, Anders S. Moderated estimation of fold change and dispersion for RNA-seq data with DESeq2. Genome Biol. 2014;15(12):550. 10.1186/s13059-014-0550-8. 25516281 10.1186/s13059-014-0550-8 PMC4302049

[22] Matsumoto T, Imagama S, Hirano K, Ohgomori T, Natori T, Kobayashi K, et al. CD44 expression in astrocytes and microglia is associated with ALS progression in a mouse model. Neurosci Lett. 2012;520(1):115–20. 10.1016/j.neulet.2012.05.048. 22634473 10.1016/j.neulet.2012.05.048

[23] McQuade A, Coburn M, Tu CH, Hasselmann J, Davtyan H, Blurton-Jones M. Development and validation of a simplified method to generate human microglia from pluripotent stem cells. Mol Neurodegener. 2018;13(1):67. 10.1186/s13024-018-0297-x. 30577865 10.1186/s13024-018-0297-x PMC6303871

[24] Nam K, Oh S, Lee K, Yoo S, Shin I. CD44 regulates cell proliferation, migration, and invasion via modulation of c-Src transcription in human breast cancer cells. Cell Signal. 2015;27(9):1882–94. 10.1016/j.cellsig.2015.05.002. 25979842 10.1016/j.cellsig.2015.05.002

[25] Pinner E, Gruper Y, Ben Zimra M, Kristt D, Laudon M, Naor D, et al. CD44 splice variants as potential players in Alzheimer’s disease pathology. J Alzheimers Dis. 2017;58(4):1137–49. 10.3233/JAD-161245. 28550248 10.3233/JAD-161245

[26] Prinz M, Priller J. Microglia and brain macrophages in the molecular age: from origin to neuropsychiatric disease. Nat Rev Neurosci. 2014;15(5):300–12. 10.1038/nrn3722. 24713688 10.1038/nrn3722

[27] Rayahin JE, Buhrman JS, Zhang Y, Koh TJ, Gemeinhart RA. High and low molecular weight hyaluronic acid differentially influence macrophage activation. ACS Biomater Sci Eng. 2015;1(7):481–93. 10.1021/acsbiomaterials.5b00181. 26280020 10.1021/acsbiomaterials.5b00181 PMC4533115

[28] Riehl TE, Santhanam S, Foster L, Ciorba M, Stenson WF. CD44 and TLR4 mediate hyaluronic acid regulation of Lgr5+ stem cell proliferation, crypt fission, and intestinal growth in postnatal and adult mice. Am J Physiol Gastrointest Liver Physiol. 2015;309(11):G874-887. 10.1152/ajpgi.00123.2015. 26505972 10.1152/ajpgi.00123.2015 PMC4669354

[29] Sakthivel PS, Scipioni L, Karam J, Keulen Z, Blurton‐Jones M, Gratton E, et al. Organelle phenotyping and multi‐dimensional microscopy identify C1q as a novel regulator of microglial function. J Neurochem. 2024. 10.1111/jnc.16173. 39018376 10.1111/jnc.16173 PMC11449638

[30] Sawada R, Nakano-Doi A, Matsuyama T, Nakagomi N, Takayuki Nakagomi T. CD44 expression in stem cells and niche microglia/macrophages following ischemic stroke. Stem Cell Investig. 2020;7:4–4. 10.21037/sci.2020.02.02. 32309418 10.21037/sci.2020.02.02 PMC7154319

[31] Schmittgen TD, Livak KJ. Analyzing real-time PCR data by the comparative CT method. Nat Protoc. 2008;3(6):1101–8. 10.1038/nprot.2008.73. 18546601 10.1038/nprot.2008.73

[32] Stevens B, Allen N, Vazquez L. The Classical Complement Cascade Mediates CNS Synapse Elimination Cell. 131:1164–1178. 10.1016/j.cell.2007.10.036 18083105

[33] Solé-Domènech S, Cruz DL, Capetillo-Zarate E, Maxfield FR. The endocytic pathway in microglia during health, aging and Alzheimer’s disease. Ageing Res Rev. 2016;32:89–103. 10.1016/j.arr.2016.07.002. 27421577 10.1016/j.arr.2016.07.002 PMC5127718

[34] Son M, Porat A, He M, Suurmond J, Santiago-Schwarz F, Andersson U, et al. C1q and HMGB1 reciprocally regulate human macrophage polarization. Blood. 2016;128(18):2218–28. 10.1182/blood-2016-05-719757. 27683415 10.1182/blood-2016-05-719757 PMC5095756

[35] Soneson C, Love MI, Robinson MD. Differential analyses for RNA-seq: transcript-level estimates improve gene-level inferences. F1000Res. 2015;4:1521. 10.12688/f1000research.7563.2. 26925227 10.12688/f1000research.7563.1 PMC4712774

[36] Stephan AH, Madison DV, Mateos JM, Fraser DA, Lovelett EA, Coutellier L, et al. A dramatic increase of C1q protein in the CNS during normal aging. J Neurosci. 2013;33(33):13460–74. 10.1523/JNEUROSCI.1333-13.2013. 23946404 10.1523/JNEUROSCI.1333-13.2013 PMC3742932

[37] Taylor KR, Yamasaki K, Radek KA, Nardo AD, Goodarzi H, Golenbock D, et al. Recognition of hyaluronan released in sterile injury involves a unique receptor complex dependent on Toll-like receptor 4, CD44, and MD-2. J Biol Chem. 2007;282(25):18265–75. 10.1074/jbc.M606352200. 17400552 10.1074/jbc.M606352200

[38] Thion MS, Low D, Silvin A, Chen J, Grisel P, Schulte-Schrepping J, et al. Microbiome influences prenatal and adult microglia in a sex-specific manner. Cell. 2018;172(3):500-516.e16. 10.1016/j.cell.2017.11.042. 29275859 10.1016/j.cell.2017.11.042 PMC5786503

[39] Vachon E, Martin R, Kwok V, Cherepanov V, Chow C-W, Doerschuk CM, et al. CD44-mediated phagocytosis induces inside-out activation of complement receptor-3 in murine macrophages. Blood. 2007;110(13):4492–502. 10.1182/blood-2007-02-076539. 17827392 10.1182/blood-2007-02-076539 PMC2234794

[40] Vachon E, Martin R, Plumb J, Kwok V, Vandivier RW, Glogauer M, et al. CD44 is a phagocytic receptor. Blood. 2006;107(10):4149–58. 10.1182/blood-2005-09-3808. 16455948 10.1182/blood-2005-09-3808

[41] Vay SU, Flitsch LJ, Rabenstein M, Rogall R, Blaschke S, Kleinhaus J, et al. The plasticity of primary microglia and their multifaceted effects on endogenous neural stem cells in vitro and in vivo. J Neuroinflammation. 2018;15(1):226. 10.1186/s12974-018-1261-y. 30103769 10.1186/s12974-018-1261-y PMC6090672

[42] Villa A, Gelosa P, Castiglioni L, Cimino M, Rizzi N, Pepe G, et al. Sex-specific features of microglia from adult mice. Cell Rep. 2018;23(12):3501–11. 10.1016/j.celrep.2018.05.048. 29924994 10.1016/j.celrep.2018.05.048 PMC6024879

[43] Wolf KJ, Shukla P, Springer K, Lee S, Coombes JD, Choy CJ, et al. A mode of cell adhesion and migration facilitated by CD44-dependent microtentacles. Proc Natl Acad Sci U S A. 2020;117(21):11432–43. 10.1073/pnas.1914294117. 32381732 10.1073/pnas.1914294117 PMC7261014

[44] Xiao Y, Yang K, Wang Z, Zhao M, Deng Y, Ji W, et al. CD44-mediated poor prognosis in glioma is associated with M2-polarization of tumor-associated macrophages and immunosuppression. Front Surg. 2022;8:775194. 10.3389/fsurg.2021.775194. 35187044 10.3389/fsurg.2021.775194 PMC8850306

[45] Yu G, Wang L-G, Han Y, He Q-Y. clusterProfiler: an R package for comparing biological themes among gene clusters. OMICS. 2012;16(5):284–7. 10.1089/omi.2011.0118. 22455463 10.1089/omi.2011.0118 PMC3339379

